# Phenolic Compounds in Food: Characterization and Health Benefits

**DOI:** 10.3390/molecules27030783

**Published:** 2022-01-25

**Authors:** Mirella Nardini

**Affiliations:** CREA, Research Centre for Food and Nutrition, Via Ardeatina 546, 00178 Rome, Italy; mirella.nardini@crea.gov.it

Oxidative stress is involved in the onset and development of several human diseases, such as cardiovascular diseases, diabetes, ageing, cancer, and neurodegenerative diseases [1]. Antioxidants from one’s diet, which counteract oxidative stress, may offer protection toward these pathological conditions. Polyphenolic compounds are by far the most abundant antioxidants in the human diet, being largely present in plant-based food and beverages [2,3]. Polyphenols play many roles in plants. They protect against stresses such as UV light, attacks from pests, and provide color to attract insects. Evidence from epidemiological studies suggest that the long-term consumption of polyphenolic-rich foods affords protection against the development of cardiovascular and degenerative diseases, cancer, and diabetes [4,5,6]. The absorption and metabolism of polyphenols have been extensively described. Polyphenols from foods and beverages are quickly absorbed and metabolized in humans. Gut microbiota play a critical role in the absorption process. For individuals who regularly consume wine, beer, coffee, and tea, these beverages represent the main sources of dietary polyphenols [7,8,9,10]. Polyphenols content in foods and beverages strongly depends on cultivation, technology processes, and transformation. This Special Issue has collected manuscripts regarding the composition of polyphenols in food, with special emphasis to extractive and analytical aspects. The role of technological processes in the nutritional quality of foods was also considered. Research studies dealing with biological activity and healthy effects of polyphenols were also presented.

The first research article of this Special Issue by Johnson et al. [11] deals with the characterization of phenolic profiles of Australian faba bean (*Vicia faba* L.) varieties. The phenolic acids and flavonoids composition of ten commercial Australian faba bean varieties grown at two different locations was presented. Phenolic profiling by HPLC revealed catechin and rutin as the most abundant flavonoids. Among phenolic acids, syringic acid was found in high concentrations. The levels of phenolics varied significantly with the varieties. Some effects of the growing location on phenolics content were also observed.

The study of El Cadi et al. [12] describes the phytochemical content and the antioxidant activity of the Moroccan species *Chamaerops humilis* L. fruits. The n-hexane fraction analyzed using GC/MS exhibited 69 compounds belonging to different chemical classes. The polyphenolic profile obtained using HPLC-PDA/MS led to the identification of 16 and 13 compounds in two different extracts obtained by ethyl acetate or methanol extraction, with ferulic acid and chlorogenic acid as major compounds, respectively. The ethyl acetate extract showed the highest antioxidant activity measured using the DPPH method. The results obtained highlighted *Chamaerops humilis* L. fruits as important sources of bioactive compounds.

Another study by Pinto et al. [13] reports the polyphenol profiling, obtained using MALDI-TOF-MS and ESI-qTOF-MS, of chestnut pericarp, integument, and curing water extracts to qualify these food by-products as a source of antioxidants. The study provides useful indications of the molecular processes associated with the traditional practice of the water curing of chestnut, which aims to prevent insect and mold development during fruit storage. This research provides a rationale to traditional processing practices on fruit appearance and qualifies the corresponding wastes as a source of bioactive compounds for other nutraceutical applications.

The review of Serna-Vazquez et al. [14] describes the latest insights into novel deep eutectic solvents (DES) for the sustainable extraction of phenolic compounds from natural sources. DES are green alternatives for the extraction processes, given their low or non-toxicity, biodegradability, and reusability. The latest studies employing DES for phenolic extraction, solvent components, extraction yield, and extraction techniques were reviewed. Moreover, the most relevant DES-based studies for phenolic extraction from natural sources and potential applications were reported.

In the research article by La Torre et al. [15], the effect of the long-term storage of Kombucha from black tea on phenolics content and radical scavenging properties is reported. Kombucha is a beverage obtained by fermenting tea with the addition of sugars. It is a highly commercialized drink produced industrially. The novel finding of this pilot study revealed that kombucha from sugared black tea can be stored at refrigerator temperature for four months. After this period, the antioxidant properties of kombucha are no longer retained.

In the article of Adebo et al. [16], the kinetic of phenolic compounds’ modification during maize flour fermentation over different fermentation times is studied. The flavonoids apigenin, kaempferol, luteolin, quercetin, and taxifolin and the phenolic acids caffeic, ferulic, gallic, *p*-coumaric, sinapic, and vanillic acids were investigated. The results obtained showed that flavonoids were significantly reduced after fermentation, while phenolic acids gradually increased in prevalence. The modification of phenolics during fermentation is compound-specific, and the modification rate depends on their forms of existence in the fermented products.

The study of Salamatullah et al. [17] describes the effect of boiling techniques on the bioactive and antimicrobial properties of basil (*Ocimum sanctum*) and rosemary (*Rosmarinus officinalis*). The duration of the boiling time has a significant influence on total polyphenols and flavonoids content and antioxidant activity. Basil showed the highest antioxidant activity, total polyphenols, and total flavonoids content when it was boiled for 5 min, while rosemary exhibited the highest antioxidant activity, total polyphenols, and total flavonoids content when it was boiled for 15 min. Rosemary extracts showed high growth inhibition against Gram-positive bacteria. Salicylic acid was the most abundant phenolic compound in the rosemary sample boiled for 5 min, while acetyl salicylic acid was the most abundant phenolic compound in the basil sample boiled for 15 min.

The review of Ambra et al. [18] analyzes the main experimental reports on the fate, accessibility and bioavailability of phenolic compounds present in cooking oils and cooked vegetables. The authors considered different cooking techniques (deep-fat frying, sautéing, roasting, air-frying, microwaving, and boiling with oil), the types of oil, and the type of food, using oil alone or in combination with vegetables and how the protective effect of phenolic compounds may be improved to counteract harmful effects of oil cooking. The study of Ambra et al. [18] indicates that incomplete and contradictory observations have been published in the last few years and suggests further research is necessary to clarify the impact of cooking techniques on phenolic compounds in oil.

The article of Demir and Agaoglu deals with the identification of bioactive compounds of artichoke (*Cynara scolymus*) powder and the characterization of its antioxidant-, antimicrobial-, and metmyoglobin-reducing activity when added to minced meat during frozen storage [19]. The phenolics and flavonoids content was determined using LC-QTOF-MS. Nineteen phenolic compounds were identified via LC-QTOF-MS, with quercetin, chlorogenic acid, luteolin, catechin, and caffeic acid as the most abundant phenolics. The antioxidant activity was measured using DPPH, FRAP, and ABTS assays, while the antimicrobial activity of artichoke extract was evaluated on five different food pathogens (*S. typhimurium*, *L. monocytogenes*, *E. faecalis*, *S. aureus*, and *E. coli*) by using the disc diffusion method. In conclusion, *C. scolymus* extract exhibited good antimicrobial and antioxidant effects, stabilized the color of minced meat, and had a significant impact on sensory characteristics during the storage period.

Another interesting review of this Special Issue by Tabari et al. [20] focuses on flavonoids as promising antiviral agents against SARS-CoV-2 infection. This work provides a comprehensive review of the benefits of flavonoids in relation to COVID-19 disease. The previously reported effects of flavonoids on five RNA viruses, including influenza, Human Immunodeficiency Virus (HIV), Severe Acute Respiratory Syndrome (SARS), Middle East Respiratory syndrome (MERS), and Ebola, were considered. Flavonoids act via direct antiviral activity (via the inhibition of viral proteases, RNA polymerase, and mRNA, virus replication, and infectivity) and indirectly through the modulation of host responses to viral infection and subsequent complications (the regulation of interferons, pro-inflammatory cytokines, and inflammatory pathways).

The research article of Shafreen et al. [21] investigates the in vitro and in silico interactions of red wine polyphenols with different serum proteins: human serum albumin, fibrinogen, glutathione peroxidase 3, and C-reactive protein. The study indicates that polyphenols from red wine can interact with the key regions of serum proteins to enhance their biological activity. Among them, rutin, resveratrol, and tannic acid showed good binding affinity. Particularly, the flavonoid rutin showed the highest binding affinity with all target proteins under study. In conclusion, red wine polyphenols possess beneficial properties that can exalt their role in clinical applications.

## Data Availability

Not applicable.
